# Agarose Cryogels: Production Process Modeling and Structural Characterization

**DOI:** 10.3390/gels9090765

**Published:** 2023-09-20

**Authors:** Raffaele Mancino, Diego Caccavo, Anna Angela Barba, Gaetano Lamberti, Alice Biasin, Angelo Cortesi, Gabriele Grassi, Mario Grassi, Michela Abrami

**Affiliations:** 1 Department of Industrial Engineering, University of Salerno, 84084 Fisciano, SA, Italy; 2 Eng4Life Srl, Via Circumvallazione 39, 83100 Avellino, AV, Italy; 3 EST Srl, Academic Spin-Off, Via Circumvallazione 39, 83100 Avellino, AV, Italy; 4 Department of Pharmacy, University of Salerno, 84084 Fisciano, SA, Italy; 5 Department of Engineering and Architecture, University of Trieste, Via Valerio 6, 34127 Trieste, TS, Italy; 6 Department of Life Sciences, Cattinara University Hospital, Trieste University, Strada di Fiume 447, 34149 Trieste, TS, Italy

**Keywords:** hydrogels, modeling, equilibrium, agarose, cryogels, rheology

## Abstract

A cryogel is a cross-linked polymer network with different properties that are determined by its manufacturing technique. The formation of a cryogel occurs at low temperatures and results in a porous structure whose pore size is affected by thermal conditions. The adjustable pore sizes of cryogels make them attractive for diverse applications. In this study, the influence of the external operational temperature, which affects the cooling and freezing rates, on the production of cryogels with 2% *w*/*w* agarose is investigated. Moreover, a mathematical model is developed to simulate the cryogel production process and provide an initial estimate of the pore size within the structure. The predictions of the model, supported by qualitative light microscopy images, demonstrate that cryogels produced at higher process temperatures exhibit larger pore sizes. Moreover, the existence of pore size distribution within the gel structure is confirmed. Finally, stress relaxation tests, coupled with an image analysis, validates that cryogels produced at lower temperatures possess a higher stiffness and slower water release rates.

## 1. Introduction

Technologies involving the use of macroporous polymeric materials are of great interest. Cryogelation (or gelation at subzero temperatures) is a promising technology that allows for the preparation of macroporous hydrogels called cryogels [1,2]. In cryogelation, there are two physical phases: a solid phase consisting of freezing solvent crystals and a liquid phase containing the polymer. Gelation occurs in the liquid phase while the crystals continue to grow, resulting in an interconnected porous structure. The main characteristics of cryogels are a high elasticity and interconnected pores. Unlike conventional gels, where the solvent is bound to the polymer network, cryogels contain both solvent in their interconnected pores and solvent bound to the polymer network [3]. Due to their highly interconnected and open macroporous structure, cryogels have quickly become essential for many biomedical applications. In particular, cryogels based on natural polymers, mostly agarose-based, have been frequently used for biological and biomedical applications because of their good biocompatibility, biodegradability, low toxicity, and other key advantages [4]. A number of unique properties of cryogel, including a high water content, considerable pore size and porosity, and high pore connectivity and consistency, make them very similar to native soft tissues [5].

Agarose-based gels are therefore mainly used to build scaffolds for tissue engineering [6,7], drug delivery applications [8,9], and are used as sieves in gel electrophoresis [10].

An estimation of the pore size in the polymer matrix could improve the efficient use of cryogels and open up new application possibilities. The size of the pores and their associated structure and mechanical properties are influenced by the polymerization and crystallization rates, which, in turn, are affected by factors such as the cooling rate and concentration of the cross-linking. However, a quantitative characterization of the texture of cryogels is challenging due to their soft nature and strong hydration. Therefore, only qualitative or semi-quantitative structural and textural analyses have been performed in the literature. Conventional approaches to such characterization often involve a microscopic examination of dried or freeze-dried cryogels using various imaging techniques, including optical microscopy, scanning electron microscopy (SEM) [11], cryo-SEM, and confocal laser scanning microscopy (CLSM) [12]. Unfortunately, these methods often require intensive sample preparation, such as solvent evaporation or freezing, which can lead to structural changes. In addition, the analytical range is often limited to a restricted region, making a comprehensive evaluation difficult [13]. In some work, researchers have used mercury porosimetry to accurately determine pore size distribution and other structural properties [14]. However, because samples must be dried, mercury intrusion porosimetry is unsuitable for analyzing highly pliable cryogels [15,16].

In light of the impact of the porosity and polymer compaction within the pore walls, evaluating the mechanical properties of cryogels becomes interesting. The compressive modulus and dynamic shear can be characterized [2,17,18]. Suner et al. [19] performed uniaxial compression tests on cryogel specimens in both equilibrium swollen and dried states and obtained data such as their elastic modulus and stress relaxation curves. In addition, the combined use of rheology and low-field NMR proved useful in determining the nano- and microstructures of porous hydrogels composed of bacterial cellulose and acrylic acid [20].

Although these methodologies are accurate, their analyses are time-consuming. Sometimes, a quick estimate of pore size is sufficient to individuate a particular cryogel for a given application. The size of the solvent crystals in an object can be estimated using mathematical models or empirical equations. For example, Arsiccio et al. [21] developed a versatile model applicable across a wide range of freezing conditions and solutions containing both amorphous and crystalline solutes.

The aim of this study is to investigate the influence of the process temperature on the structural properties of cylindrical agarose cryogels. An in-depth morphological analysis is out of the scope of this study, as Yokoyama et al. [22] extensively investigated this aspect using polarized optical microscopy and SEM techniques on 1D-like (cylinders with length/radius ≫ 1) agarose cryogels. Their studies showed that directional freezing leads to the crystallization of isolated ice crystal phases, resulting in the formation of a honeycomb-like gel phase with an increased polymer content. The central hypotheses of this study are that the average size of these ice crystals can be modulated by varying the cryogel production temperature, and that a distinct pore size distribution exists within the cryogel. Furthermore, these variations in pore size are expected to manifest themselves in different poromechanical behavior. To test these hypotheses, a mathematical model of the production process is developed that takes into account the dimensions of the solvent crystals. In addition, mechanical stress relaxation tests are used in conjunction with a tailored image analysis technique to quantify the axial stresses, deformations, and water release in the samples prepared at different temperatures.

## 2. Results and Discussion

### 2.1. Cryogel Formation

The temperature profiles were recorded during the preparation of the cryogels. These profiles were recorded using three thermocouples placed along the radii of the specimens, capturing data for three different cooling rates. A maximum temperature of −12 °C was chosen to ensure the attainment of the crystallization temperature, denoted as $T_{c}$, which was experimentally determined to be around −8 °C. To avoid damage to the refrigeration machine (chiller), the lower threshold temperature was set at −25 °C to prevent the aqueous glycol solution (the fluid thermal bath) from freezing. As a midpoint, a temperature of −19 °C was selected for the third set point.

**Figure 1:**
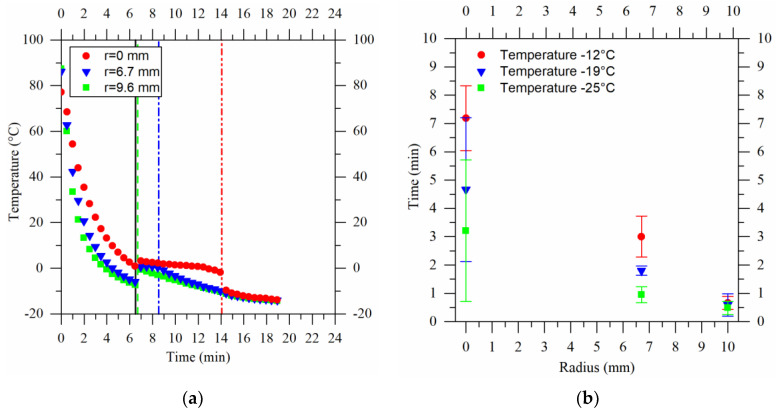
(**a**) Temperature profile of a cryogel produced at −19 °C. The curves were obtained using three thermocouples positioned at the center of the sample and at 6.7 mm and 9.6 mm from it. The distance of the probes from the base of the cryogel was approximately 1 mm. Between the vertical lines the freezing phase is shown, which was used for the calculation of freezing times. (**b**) The freezing time for cryogels produced at temperatures of −12 °C, red dots, −19 °C, blue triangles, and −25 °C, green squares.

Figure 1a shows the temperature profile of a cryogel prepared at a temperature of −19 °C. The three curves represent the measurements of the thermocouples placed within the cryogel at different distances from the center—namely, 0 mm, 6.7 mm, and 9.6 mm. The *x*-axis represents the time elapsed since the beginning of the experiment, indicating the point of contact between the cryogel and the thermal bath. The *y*-axis, on the other hand, shows the temperature measurements recorded by the thermocouples. In the initial two minutes, a rapid decrease in temperature to around 20 °C was evident. At this point, a sol–gel transition occurred (below 35 °C for the agarose used); therefore, it cannot be ruled out that this transition was completed before the formation of the solvent crystals. It is plausible that the agarose gel could initially form as a standard gel and subsequently undergo water crystallization to create the cryogel. Upon reaching the initiation temperature for crystallization, a temperature jump transpired (induced by the heat release from nucleation [23]) and the crystals began to grow.

The vertical solid black line in Figure 1a is positioned at the beginning of the freezing phase, whereas the vertical dashed lines are positioned at the end of the cryogel freezing phase at the specific radius. Beyond the last vertical line dashed line, the entire sample transitioned into the solid phase, and the temperature started to decrease again. The time interval between the black line and another gives an indication of the freezing time, which is calculated as:

(1) $$t_{c}=t_{cf}-t_{ci}$$

where $t_{ci}$ represents the starting time of the freezing and $t_{cf}$ represents the ending time of the freezing. Examining the graphs (b) in Figure 1, it is possible to observe the average freezing times for various process temperatures and distances from the center of the cryogel. Near the wall of the mold (points at a radius of 9.6 mm), rapid freezing occurred in all cases, lasting less than a minute. To freeze one third of the sample, only 0.94 min was needed at −25 °C (green square at 6.7 mm), while longer durations were needed as the process temperature increased. This is even more evident looking at the result obtained at the center of the sample. In terms of the standard deviation, a noteworthy observation is the relatively large value at the center. This phenomenon is attributed, as explained later, to the probe’s positioning during the experiments.

### 2.2. Process Temperature Modeling

**Figure 2:**
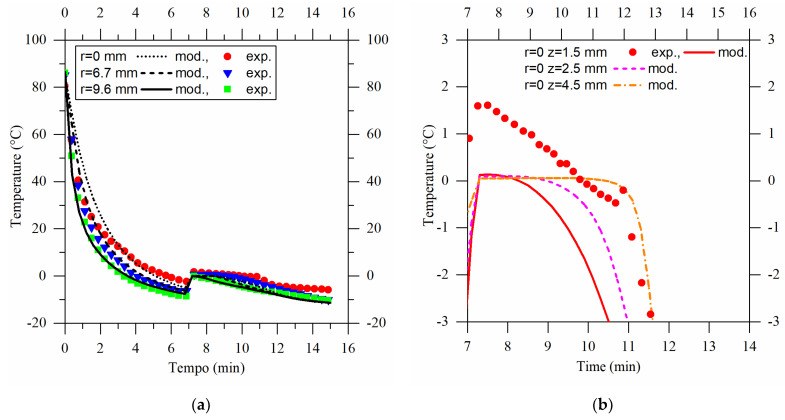
(**a**) The experimental water temperature profile compared with the modeling profile obtained with a process temperature of −19 °C. Symbols show experimental results, lines show model results. Green squares show the temperature profile near the mold, while blue triangles show the one at 6.7 mm from the center of the sample, and red dots show the one in the center. (**b**) The modeling results, represented by lines, are obtained at the center of the sample, but at a different probe z distance from the bottom surface. Symbols show the experimental results obtained at the center of the sample.

To validate the accuracy of the developed first-principle model, it was implemented and numerically solved using the finite element method in COMSOL Multiphysics 5.5 (Burlington, MA, USA), and then compared with the experimental results. The experimental data obtained for water at −19 °C were compared with the predictions of the model. Importantly, it should be emphasized that the model did not require any adjustable parameters; instead, it only required the specification of the operating conditions (i.e., the process temperature). The temperature of −19 °C was chosen to verify the accuracy of the model. A comparison of the experimental results with the model predictions is shown in Figure 2a.

The three curves in the model, represented by lines, were obtained by using three temperature “probes” within the modeling domain. These probes were positioned at the same locations as the thermocouples used in the experimental setup, i.e., at the center of the sample and at distances of 6.7 mm and 9.6 mm from it. The first-principle model was used to predict the experimental outcomes acquired using the three K-type thermocouples, whose accuracy was ±1 °C. The predictions of the model agreed well with the experimental results for the thermocouple situated near the mold wall and closely resembled those at r = 6.7 mm, especially before the phase transition. During the freezing phase, however, the red model curve (r = 0) significantly diverged from the experimental data. This discrepancy arose from the height positioning of the central thermocouple. Slight variations in the distance between the thermocouple and the base of the sample, even within a few millimeters of the hypothesized value (1.5 mm), led to considerable changes in the freezing times, spanning several minutes. In Figure 2b, the modeling temperature evolution of the sample at the center (r = 0) is illustrated using lines, representing different height positions during the freezing phase. Changes in the thermocouple position in the model led to longer predicted freezing times. Hence, the model demonstrated its capacity to predict the freezing process without the need for optimization parameters. This enables its applicability in describing the production process of agarose cryogel.

### 2.3. Porosity Estimation of Cryogels

The model implemented in COMSOL Multiphysics facilitated the direct prediction of various parameters, including the freezing duration, temperature distributions, fluid velocities within the system, and the temporal evolution of the material properties. Some of these data were used to estimate the size of the ice crystals. Indeed, according to Sman et al. [24], the size of these crystals can be estimated using a simple empirical equation that was obtained by fitting the literature data from a certain number of materials; although agarose cryogels were not among them, some with similar characteristics were present.

(2) $$d_{p}=50v_{c}^{-0.25}$$

where $d_{p}$ represents the diameter of the solvent crystals in micrometers (µm) and $v_{c}$ signifies the freezing rate in degrees Celsius per second (°C/s). In this work, “$v_{c}$” was computed as the time derivative of the temperature d(T/t) when the value of the volume fraction of the solid phase $\theta_{ph1}$ was between 0.96 and 0.99, indicating the near-complete freezing of the gel. This equation, once implemented into COMSOL, provided a rapid initial estimate of the crystal size within the agarose cryogels.

**Figure 3:**
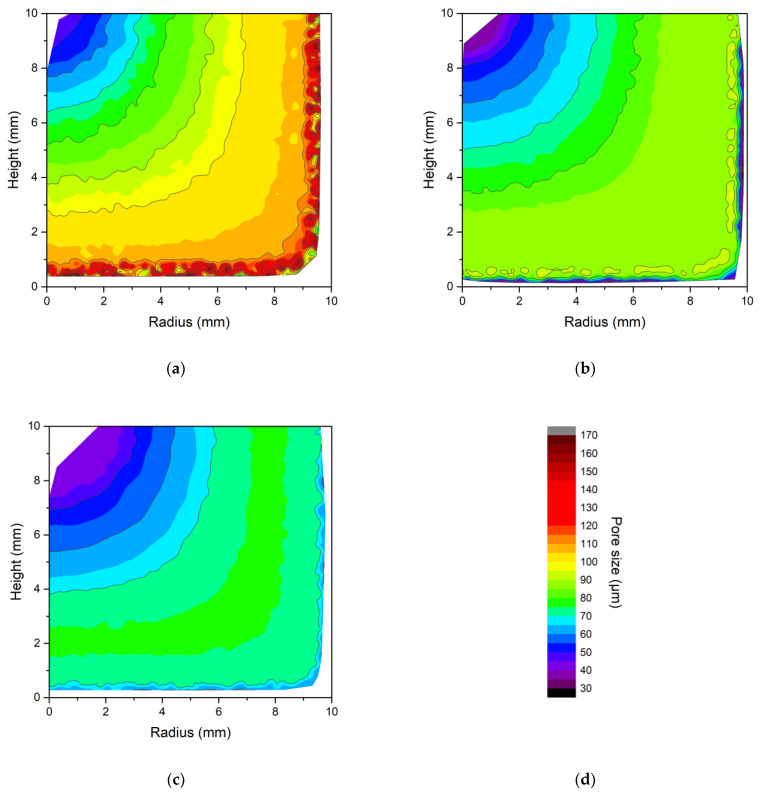
(**a**) The estimated pore size within agarose cryogels. The graphs show the sample radius on *x*-axis and the height on *y*-axis; the pore size is shown on a color scale. (**a**) was obtained using model data by setting a bath temperature of −12 °C. (**b**) shows the values obtained with a temperature of −19 °C, while (**c**) shows the pore size for a process temperature of −25 °C. (**d**) shows the color bar to relate the color to the pore size in μm from 30 to 170.

The solvent crystal sizes estimated using the model data and Equation (2) are shown in Figure 3. Here, the pore size is shown on a chromatic scale, in relation to the spatial coordinates, height, and radius of the sample. From “a” to “c”, the results for cryogels produced at −12 °C, −19 °C, and −25 °C are shown, respectively. Regardless of the process temperature, near the walls, when crystallization began, the cooling rate was very high and the pore diameter was small. Moving away from the walls, the pore diameter increased until it reached a maximum value where the cooling rate was at minimum, because the solvent crystals had more time to grow. From here, as one moved toward the center of the cryogel, the volume of liquid to be frozen decreased, so the cooling rate increased. As a result, the diameter of the solvent crystals would be smaller near the walls and in the center of the sample. From the data shown in Figure 3, the volume-averaged pore diameters at different process temperatures were also calculated: $\langle d_{p}\rangle =\iint \left(2\;\pi \;r\;d_{p}\left(r,z\right)dr\;dz\;\right)/\iint \left(2\;\pi \;r\;dr\;dz\right)$. At the lowest temperature (−25 °C), an average diameter of 69 μm was estimated, 83 μm for a temperature of −19 °C, and 97 μm for that of −12 °C.

**Figure 4:**
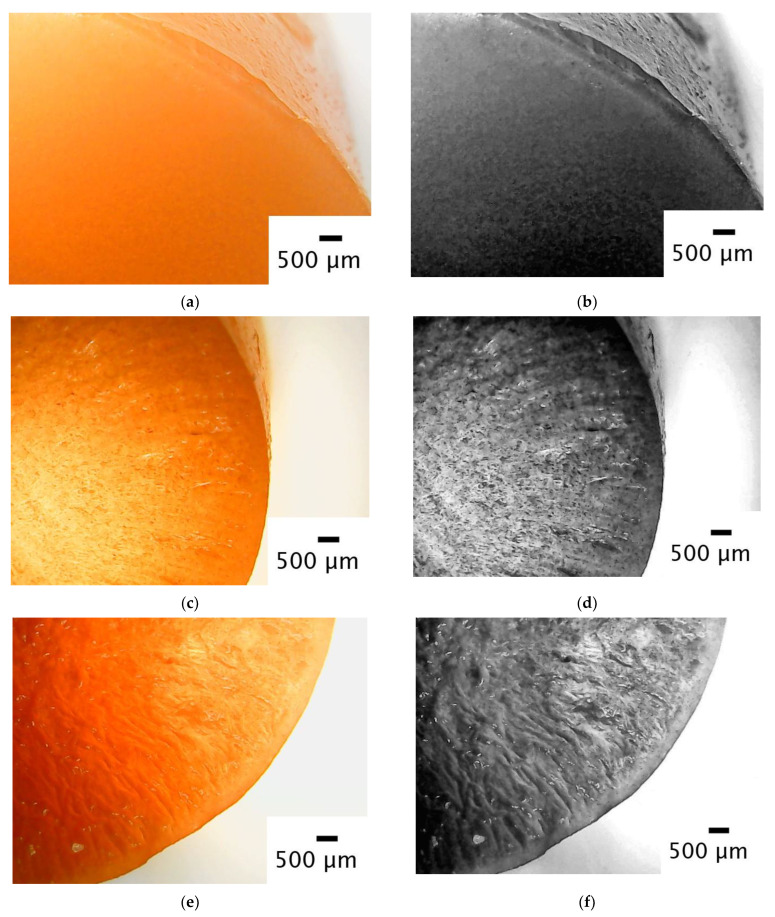
(**a**) The photo of a 2% *w*/*w* agarose hydrogel dyed with iron oxide obtained using a light microscope (**a**) and the same photo on grayscale (**b**). Two cryogels dyed with iron oxide produced at a temperature of −25 °C (**c**) and −12 °C (**e**) photographed with a light microscope and their respective grayscale photos (**d**,**f**).

In this study, light microscopy was utilized solely for a qualitative assessment. A primary morphological characterization was not the focus of this work, as this has been comprehensively conducted elsewhere [22]. As such, light microscopy images were included to provide a qualitative confirmation of the predictions of the model. The images allowed for a visual comparison of the structural attributes among a hydrogel and two cryogels that were dyed with ferric oxide (Figure 4). The hydrogel (Figure 4a,b) showed a smooth surface, and no macroscopic pores were observable through light microscopy. It is worth noting that agarose hydrogels are known to possess nanopores, as reported in the literature [25,26]. However, the cryogels produced at −25 °C (Figure 4c,d) and −12 °C (Figure 4e,f) had larger pore sizes. The cryogels displayed channels of a macroscopic size that were likely formed due to ice crystal joining, as it can be seen in Figure 4c,d and even better in Figure 4e,f. Indeed, the channels, whose size was related to the porosity of the system, were clearly visible in the cryogel produced at −12 °C, less so in the cryogel produced at −25 °C, and were entirely absent in the hydrogel. The fusion of the ice crystals was not predicted by the proposed model, but it did predict that the largest solvent crystals were located between the center and side surfaces of the sample. Indeed, upon observation near the edges (Figure 4c–f), pores were not discernible, as they were on the nanometer scale. However, as one progressed towards the center of the gel, channels of a macroscopic size were observed, passing through a maximum (especially visible in Figure 4c,d).

From the images in Figure 4, it is therefore possible to state qualitatively that, as shown by the modeling result in Figure 3, the pore size decreased as the process temperature decreased and, as in the built model, the maximum pore size was between the center and the edges of the samples.

### 2.4. Mechanical Testing and Image Analysis

**Figure 5:**
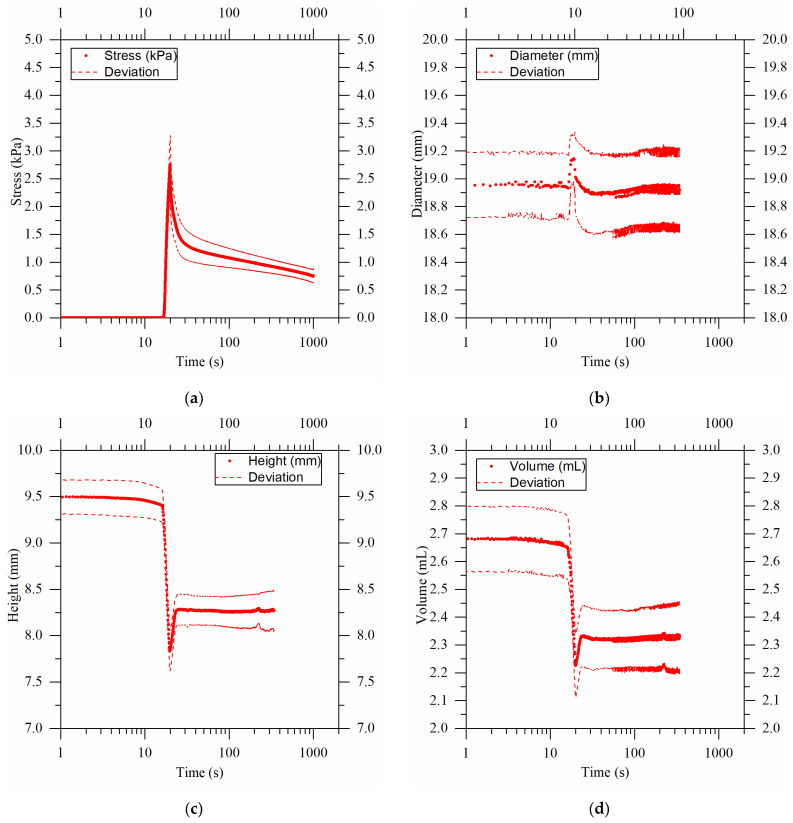
Results obtained from unconfined compression tests coupled with image analysis for 2% *w*/*w* agarose cryogels produced at a temperature of −12 °C. (**a**) shows the stress value, solid line, as a function of time, setting a final strain of 10%. The dashed lines represent the standard deviation from the mean of the analyzed samples. In (**b**), the average diameter change obtained through image analysis is shown. In (**c**), the change in height during the compression test is given, and the minimum point is given by the release of water from the samples. In (**d**), the sample volume changes from the height and diameter data obtained from image analysis are shown.

Unconfined stress relaxation tests were carried out to establish a correlation between porosity and structure. During the mechanical tests, the photos taken were analyzed to obtain the changes, over time, in the height and diameter of the sample and in the volume variation (calculated). Figure 5 illustrates the time evolution of the stress, diameter, height, and volume of the sample. Average values are reported by dots, while dashed lines depict the standard deviation around the mean.

Figure 5a shows the value of the stress over time, setting a final strain of 10% for the cryogels produced at a temperature of −12 °C. During the first few seconds, the stress curve maintained a constant value of zero because the texture analyzer probe had not yet come into contact with the surface of the cryogel. Thereafter, the curve increased rapidly until it reached a peak value at the 10% strain of 2.7 kPa. Immediately after, the stress decreased due to the release of water by the cryogels (which already occurred during sample deformation) and the relaxation of the chains. Figure 5b shows the diameter increase upon compression, while it came back to the original value during the relaxation step. The graph in Figure 5c allows for observing the change in the sample height during the test. A minimum point near 20 s was observed, coinciding with the instant when the set point strain value was reached. Here, the release of water by the cryogel created a “shadow zone” that interfered with the image analysis. A few moments later, the water left the field of view of the camera and the actual height value was given. The last curve in Figure 5d shows the volume. It was obtained from the height and diameter values seen above. Its trend was similar to that of the height, and a volume decrease of about 0.4 mL was observed for the cryogels produced at −12 °C.

**Figure 6:**
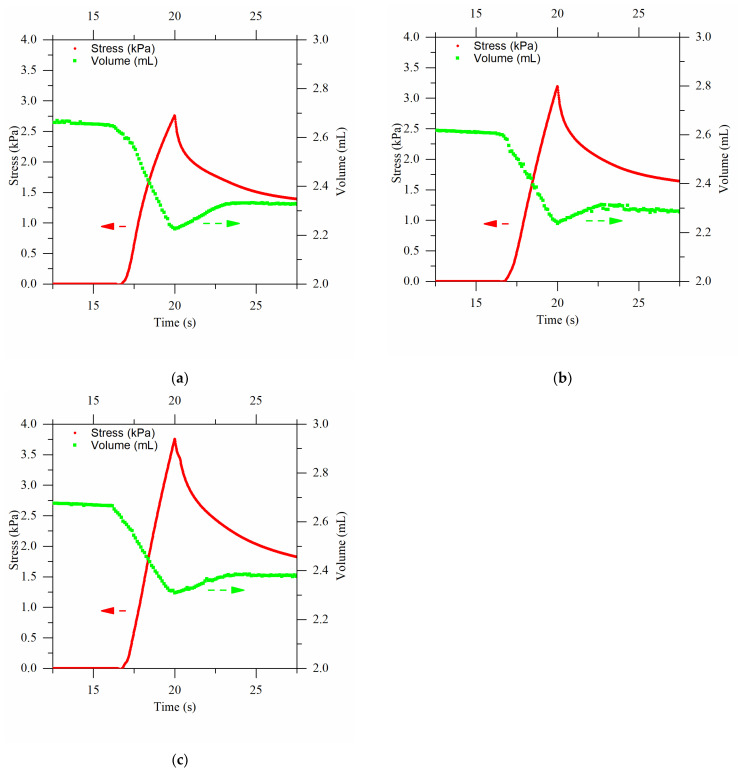
On the same graph: the stress curve is in red and in the volume change curve is in green during the unconfined compression test with 10% imposed strain. Arrows help to read the data on the proper y-axis. In (**a**), the curves obtained for cryogels produced at a bath temperature of −12 °C are shown, in (**b**) the curves for products obtained at −19 °C, and in (**c**) those for a temperature of −25 °C.

A difference between the cryogels can be observed in Figure 6a–c, showing the stress (red curve) and volume (green squares) changes occurring upon compression, in the cases of cryogels produced at −12 °C, −19 °C, and −25 °C, respectively. Comparing the stress curves for the different process temperatures, an interesting finding can be highlighted. The cryogels produced at lower temperatures exhibited a higher peak stress value of 3.7 kPa, because of their stiffer structure. Increasing the process temperature resulted in softer cryogels. This shows how the process temperature affected the structure of the product and was partly attributable to the pore size. As noted earlier, the cryogels produced at −25 °C had smaller pore sizes, thus correlating to a stiffer structure. After all, the hydrogels at 2% *w*/*w* agarose, whose pores were nanometer in size, exhibited a peak stress value of about 19 kPa for a strain of 10% [8]. Therefore, producing cryogels at temperatures below −25 °C, possibly using liquid nitrogen, could potentially lead to further increases in their maximum strain value, approaching that of a homogeneous hydrogel when nanometer pore sizes are achieved. By comparing the stress curves, it is evident that they exhibited different slopes during the compression phase: the higher the cryogel process temperature, the lower the slope. Indeed, in the cryogels produced at −12 °C, the section of the curve that characterized compression was curvilinear, while it assumed an initial linear trend followed by bending near the peak value in the case of the gels produced at −19 °C. In contrast, the same section of the curve in the cryogels at −25 °C appeared straight. This was due to the release of water during compression, which reduced the stress response provided by the sample. Thus, the release of water was faster in the cryogels with larger pores, i.e., those produced at higher temperatures. This water release was also confirmed by the volume variation, which was the same at equilibrium in all three cases (around 0.4 mL), but with different kinetics. Indeed, in this case, the slope (negative) was higher for the cryogels produced at a higher temperature (with larger pores). Therefore, the combination of the mechanical analysis and image analysis confirmed a different structure in the cryogels produced at different temperatures and highlighted the impact of the structure on the macroscopic properties, such as the poroelastic behavior of the system.

## 3. Conclusions

In this work, the influence of the production temperature on the structural characteristics of 2% *w*/*w* agarose cryogels was investigated. A first-principle model was developed to analyze the cryogel production process. The process involved freezing a polymer solution containing agarose within a glycol-based thermal bath.

During production, temperature monitoring was conducted using three K-type thermocouples. Cryogels were produced with bath temperatures of −12 °C, −19 °C, and −25 °C. The temperature profiles inside the gels obtained from these experiments provided insights into the average crystallization initiation temperature, phase transition, and duration needed for complete cryogel freezing. The data collected during this stage served as the basis for constructing a predictive model capable of estimating cryogel freezing times for varying process temperatures and providing an initial assessment of the pore size in a product. However, it should be noted that further investigations are required to fully replicate the intricate channel structure observed in cryogels.

Both the model predictions and qualitative microscope images highlighted that the cryogels produced at higher process temperatures exhibited larger pore sizes. Furthermore, it was conclusively established that pore size distribution existed within the gel structure.

Prompted by these significant findings, further investigation was conducted using unconfined mechanical stress relaxation tests, in conjunction with an image analysis. These tests revealed that the cryogels produced at lower temperatures possessed a higher stiffness and slower water release rates.

In summary, the developed mathematical model, in combination with the experimental investigations, effectively confirmed the central hypotheses of this study. The research provides a clear understanding of how the cryogel production temperature influences the ice crystal size, pore size distribution, and subsequent poromechanical behavior, contributing valuable insights to the field of cryogel engineering.

## 4. Materials and Methods

### 4.1. Materials

Agarose was supplied by Sigma-Aldrich (Milan, Italy) (CAS 9012-36-6). As the refrigerant fluid, a 40% *w*/*w* aqueous glycol solution produced by S.I.RA.L. S.p.A. (Nola, Italy) and sold as Siroil High fluidity ANTIFREEZE −20 °C was used. The aluminum molds and polylactic acid (PLA) cap were produced in the laboratory specifically for the production and testing of polymeric cryogel. Ferric oxide (CAS 1309-37-1) from Sigma-Aldrich (Milan, Italy) was used as a dye for the light microscopy analysis.

### 4.2. Production Method of Agarose Gels

The agarose was dissolved in deionized water, by it mixing and heating it at 85 °C, to obtain a 2% *w*/*w* agarose solution. Simultaneously, the aluminum molds were heated at 95 °C on a heating plate. This step was necessary to decelerate the sol–gel transition, ensuring its occurrence within the cooling equipment, Labo^®^ SM3 C300-H23. Subsequently, the prepared agarose solution was poured into the preheated molds, which were immediately positioned within the cooling apparatus (chiller). Here, the mold was placed on an upside-down beaker so that it was partially immersed in the glycol bath (a 2D axisymmetric schematic of the experimental setup is represented by the modeling domain shown in Figure 8, which accurately replicates the experimental arrangement).

After a duration of 20 min, the agarose cryogels were extracted from the molds. Excess frozen material protruding beyond the mold boundaries was excised using a cutter, and the remaining segments were allowed to thaw at room temperature for 30 min. Finally, the samples were left in water for at least 15 min to achieve equilibrium in a pure water environment, prior to their utilization. No evidence of swelling was observed using this procedure.

Iron oxide at 0.01% *w*/*w* was added to the aqueous solution to create the cryogels analyzed using light microscopy. The same procedure used for the cryogels was followed for the agarose hydrogel, with the distinction that the hydrogel was allowed to gel at room temperature.

### 4.3. Thermal Profiles during the Formation of Cryogels

The temperature of the cryogels during their production was monitored using three K-type thermocouples. The signal from the thermocouples was amplified using the MAX31856 from Adafruit^®^. The thermocouples were inserted into the sample using a PLA cap, specifically designed and 3D printed for the application. The first thermocouple was placed in the center and the other two at 6.7 mm and 9.6 mm distances from it. The digital signal from the amplifier MAX31856 was acquired through an Arduino Mega 2560 connected to a PC, with a sampling interval of 1.5 s. The temperature profiles were recorded using MATLAB^®^ (R2021b) software.

### 4.4. Mechanical Test and Image Analysis

The mechanical tests were carried out simultaneously with the samples’ image captures. The TA.XT Plus, Stable Micro Systems Ltd. (Godalming, UK) texture analyzer was used for the mechanical tests. Unconfined stress relaxation tests were carried out at room temperature for a duration of 15 min, imposing a probe distance from the cryogel support surface of 15 mm, a compression rate of 0.3 mm/s, a trigger force of 0.01 N, and a strain of 10%. The initial diameter of the sample, obtained via the image analysis, was used to calculate the stress, as the results were acquired in terms of force. For the image analysis, photos were taken, one every 109 ms for the first 6 min of the unconfined stress relaxation test, with the Chameleon3 (CM3-U3-13S2M-CS) camera with a Fujinon HF25HA-1B lens, bought from Teledyne FLIR LLC (Wilsonville, OR, USA). The images were processed to obtain the variations in the height, diameter, and volume of the cryogels when subjected to deformation. The images were managed with a code implemented in MATLAB^®^ (R2021b).

**Figure 7:**
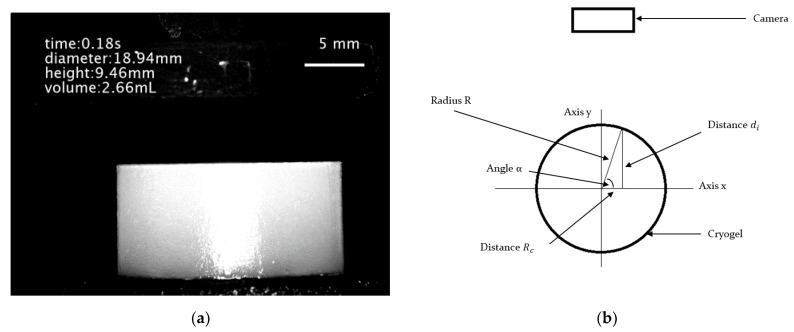
(**a**) Photograph of a cryogel during an unconfined compression test. Time, height, diameter, and volume data were obtained by processing the shot with MATLAB^®^. (**b**) Top-view schematization of cryogel and camera during unconfined compression test.

Figure 7a shows the result obtained after processing a photo. To obtain the dimensions of the cryogel, it was necessary to fix the distance between the sample and the camera in order to obtain a conversion factor of pixels to millimeters from the shot. The conversion factor (px/mm) was obtained at the plane of symmetry of the gel parallel to the camera (on the *x* axis, when y = 0, in Figure 7b and at different distances from the plane of symmetry toward the camera lenses). It was therefore possible to obtain a conversion factor function of the distance between the object and the lenses. Exploiting the conversion factor at the plane of symmetry, it was possible to obtain an average radius (or diameter), averaged on the values obtained at different heights of the sample. To instead obtain the average height of the sample, averaged on the heights calculated at different radii, the fact that a non-telecentric lens was used, along with the cylindrical geometry of the sample, was exploited. At a certain distance from the center (R_c_ in Figure 7b), knowing the total radius (R), it was possible to calculate the distance (d_i_) from the point of interest and the plane of symmetry (*x* axis) of the gel. Therefore, it was possible to use the right conversion factor, knowing exactly the distance of the point from the camera lenses.

## 5. Modeling

### 5.1. System Description

To understand the transport phenomena taking place, the system was mathematically modeled. In the cooler, there was a thermal glycol bath in which an upside-down beaker was immersed. A cylindrical aluminum mold, at a temperature of 95 °C, was placed on the beaker and partially immersed in the thermal bath. Inside the mold, the agarose solution at 85 °C was present. The thermal bath in contact with the sample was at a temperature two degrees higher than that set on the cooler, and the air in contact with it was at a temperature of 10 °C. The wall separating the room inside the cooler from the outside had a temperature of 15 °C. During the production of the cryogels, only the air and the thermal bath could move within the system. The thermal bath, despite the presence of the pump inside the cooler, did not exhibit forced convection phenomena. By exchanging energy with the hot mold, the air heated up and moved rapidly upwards due to the change in its density. Therefore, it was decided to work on a model capable of considering not only the energy exchange, but also the fluid dynamics, in order to consider the natural convection in both fluids. All the temperature values of the system, as previously mentioned, were experimentally determined.

**Figure 8:**
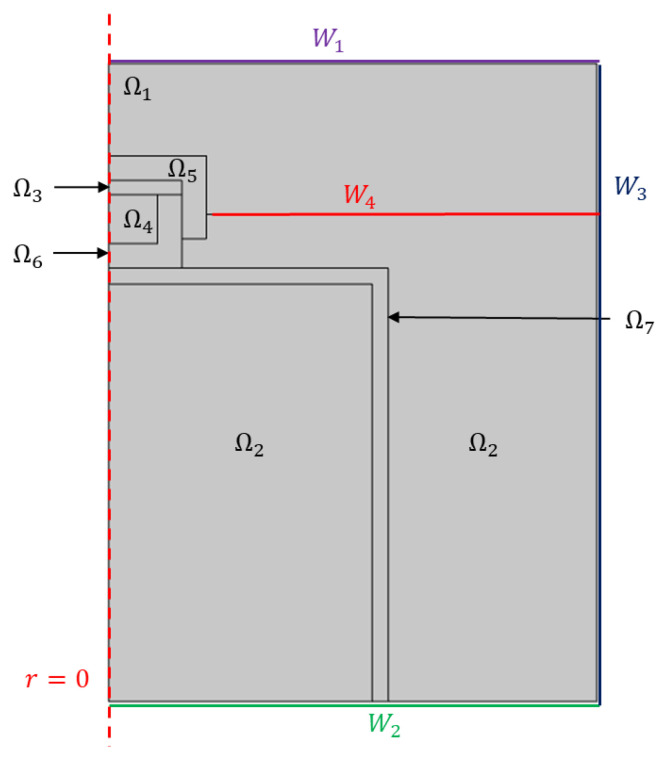
The geometry of the model. At r = 0 the axis of symmetry is fixed. $W_{1}$ is the top wall of the cooler; $W_{2}$ is the bottom wall of the cooler; $W_{3}$ are the lateral walls of fluids, which are considered adiabatic; and $W_{4}$ is the interface wall between the air and the refrigerant. $\Omega_{1}$ and $\Omega_{3}$ are the domains of air; $\Omega_{2}$ is the domain of the glycol solution, i.e., the thermal bath; $\Omega_{4}$ is the domain of the cryogel or water; $\Omega_{5}$ is the domain of PLA; $\Omega_{6}$ is the domain of aluminum mold; and $\Omega_{7}$ is the domain of the beaker.

The geometry of the system is shown in Figure 8 in 2D axisymmetric geometry. The radius *r* = 0 was matched to the axis of symmetry. The domains in the figure represent the objects in the system. The PLA cap $\Omega_{5}$ had a height of 17 mm, a thickness of 5 mm, and a radius of 15 mm; the agarose solution $\Omega_{4}$ was contained in a 10 × 10 mm (r × z) domain inside the aluminum mold $\Omega_{6}$; and the beaker $\Omega_{7}$ had a thickness of 3.3 mm and a radius of 54 mm. The glycol bath $\Omega_{2}$ had dimensions 10 × 10 cm (r × z) and 3.1 cm of air, and $\Omega_{1}$ and $\Omega_{3}$ separated it from the upper surface of the cooler.

### 5.2. Properties of Materials

The data on the PLA were retrieved from the literature [27]. The density was considered to be constant and equal to 1252 kg/m^3^. Specific heat data, as a function of temperature, were acquired through linear interpolation, extrapolating values down to 55 °C; below this threshold, the data were assumed to remain constant. The same was conducted for conductivity below 48 °C. The conductivity and dynamic viscosity properties of the 40% *w*/*w* aqueous solution of glycol, i.e., the thermal bath, were obtained down to −10 °C from the work of Bohne et al. [28]. For temperatures below −10 °C, data from “The Engineering ToolBox” website were interpolated [29]. From the same reference, specific heat and density data were obtained. Perry’s handbook [30] was used for the properties of air and water. In the sub-cooling zone, the liquid water properties were considered to be constant. The ice properties were obtained via the interpolation of the data from the aforementioned website [29]. For the aluminum, the density and conductivity were considered to be constant, with the former being equal to 2700 kg/$m^{3}$ and the latter being equal to 4.007 W/(K cm) [31]. The specific heat equation was obtained through the work of McBride et al. [32]. The properties of Pyrex glass were obtained from various papers in the literature [33,34,35].

### 5.3. Model Equations

To describe the system, the equations of motion were coupled to the energy equations. Looking at Figure 8, the laminar motion in $\Omega_{2}$ and $\Omega_{3}$ is described with the equations:

(3) $$\rho \frac{\partial u}{\partial t}+\rho \left(u\cdot \nabla \right)u=\nabla \cdot \left[-pI+K\right]+\rho g$$

(4) $$\frac{\partial \rho }{\partial t}+\nabla \cdot (\rho u)=0$$

where u is the velocity, ρ is the fluid density, p is the pressure, and t is the time. All the variables appearing in the equations are also reported in the notation table (Table 1). The energy balance was coupled to the previous equations:

(5) $$\rho C_{p}\frac{\partial T}{\partial t}=-\rho C_{p}u\cdot \nabla T-\nabla \cdot q$$

where $C_{p}$ is the specific heat of the fluid, T is the temperature, and q is the conductive heat flux. The Reynolds-Averaged Navier–Stokes (RANS) model of the low Reynolds k-ε model was used to describe the turbulent motion in $\Omega_{1}$, with k turbulent kinetic energy and ε turbulent dissipation [36]. The RANS model was coupled to the energy balance in Equation (5).

In $\Omega_{5}$, $\Omega_{6}$, and $\Omega_{7}$, as solids, only the conductive term was present in the energy balance:

(6) $$\rho C_{p}\frac{\partial T}{\partial t}=-\nabla \cdot q$$

The cryogel domain, or water $\Omega_{4}$, was also modeled as a solid, but a phase transition occurred in it. This phase transition was modeled as a function of $\theta_{ph1}$, the volume fraction of the solid phase during the transition. For $\theta_{ph1}=1$, the phase was solid, and for $\theta_{ph1}=0$, the phase was liquid ($\theta_{ph2}=1-\theta_{ph1}$ represents the liquid phase). During the transition, the properties of the cryogel were therefore a function of θ:

(7) $$C_{p}=\frac{1}{\rho }\left(\theta_{ph1}\rho_{ph1}C_{p,ph1}+\theta_{ph2}\rho_{ph2}C_{p,ph2}\right)+\lambda$$

(8) $$\kappa =\theta_{ph1}\kappa_{ph1}+\theta_{ph2}\kappa_{ph2}$$

(9) $$\rho =\theta_{ph1}\rho_{ph1}+\theta_{ph2}\rho_{ph2}$$

where λ is the latent heat due to the phase change and the subscripts “ph1” and “ph2” denote the solid and liquid phases, respectively. In addition, before the water passed into the solid phase, the temperature dropped to below 0 °C, only to return to the transition temperature when the ice crystals began to form. To model this phenomenon, the term $Q_{n}$ was added to the energy balance, as suggested by Nakagawa et al. [23].

(10) $$Q_{n}(t)=\lambda k_{i}(T_{f}-T^{*}(t))$$

where $T_{f}$ is the temperature of the freezing front, $T^{*}$ is the temperature of the subcooled solution, and $k_{i}$ is a constant that considered the nucleation rate, expressed as kg/${(m}^{3}sK)$. As was observed experimentally, the nucleation rate was high enough so that all the released heat, due to the nucleation phenomenon, was recorded by the thermocouples between two successive samplings. Therefore, modeling $k_{i}$ was fixed to the value of 430 kg/${(m}^{3}Ks)$, as in Nakagawa et al. [23], to assure this high heat release rate. Unlike Nakagawa et al. [23], who suggested that $Q_{n}$ is supplied at a fixed time, in this modeling approach, heat was supplied when the average crystallization start temperature, $T_{c}$, was reached. This temperature was calculated experimentally and was equal for both the water and agarose cryogels to about −8 °C.

**Figure 9:**
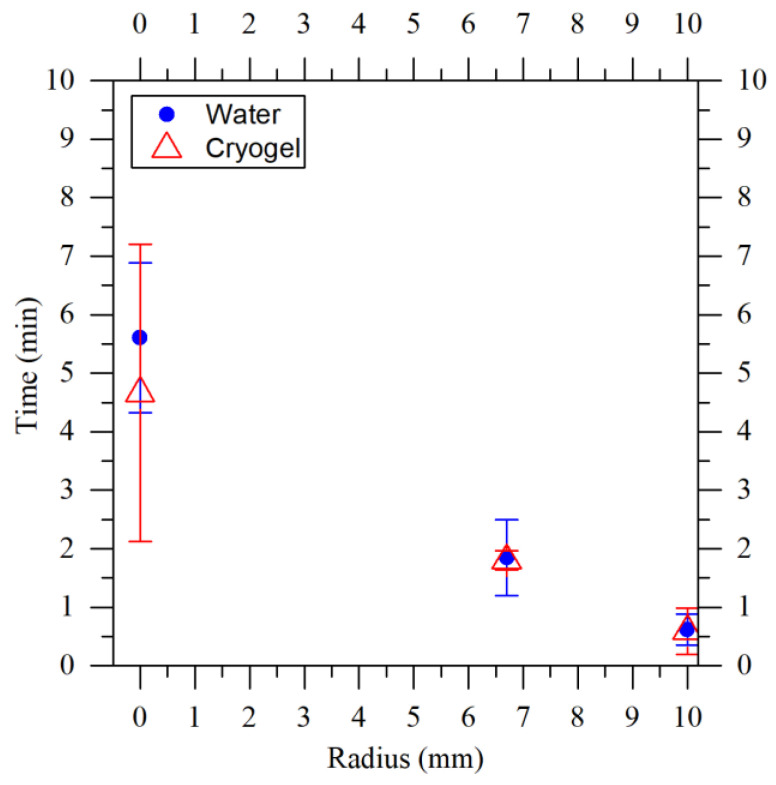
Comparison of average freezing times between water, blue dot, and cryogel, red triangle, produced at −19 °C with thermocouples placed at approximately 1 mm from the base of the mold. The bars show the deviation from the mean of the samples analyzed.

The energy balance in the domain $\Omega_{4}$ was therefore:

(11) $$\rho C_{p}\frac{\partial T}{\partial t}+\nabla \cdot q=Q_{c}+Q_{n}y(T_{inter})$$

where $y(T_{inter})$ is a rectangular function, different from 0 (and equal to 1) in the temperature range between −8 °C and 0 °C, used to control when the nucleation heat had to be released. In particular, the modeling of the system was completed with the boundary conditions and assumptions:

- looking at the boundary at r = 0, for each z, the axisymmetric condition was applied;
- a “Slip” condition was applied to the $W_{4}$ interface, so $u_{air}{=u}_{bath}$; while on all other walls, u = 0;
- the initial velocity of the fluids was zero;
- the $W_{3}$ walls were adiabatic;
- a constant temperature of 15 °C was set on the $W_{1}$ wall and a constant temperature equal to the set point of the cooler, $T_{w_{2}}$, was set on the $W_{2}$ wall;
- the known initial temperature, or the one measured at the start of the experiment, was set on the domains: $T_{\Omega_{1}}$ = 10 °C, $T_{\Omega_{2}}=(T_{w_{2}}$ + 2 °C), $T_{\Omega_{3}}$ = 25 °C, $T_{\Omega_{4}}$ = 85 °C, $T_{\Omega_{5}}$ = 25 °C, $T_{\Omega_{6}}$ = 95 °C, and $T_{\Omega_{7}}=T_{\Omega_{2}}$;
- on the bottom of the chiller, a specific constraint was imposed on the pressure, specifying that it was constant and equal to the hydrostatic pressure p=ρg·h, where g is the gravitational acceleration and h is the height;
- crystallization could not take place until the average crystallization start temperature was reached. This statement was appropriate because the temperature at which the crystallization began depended on many factors; it was not always the same and was difficult to predict. However, it is also true that, provided that only the operating conditions of the system were changed, it was possible to identify a temperature range in which crystallization occurred and hence the definition of $T_{c}$;
- the properties of the agarose cryogels were the same as water. Since the aim was to model the cryogel production process and the cryogels produced were 98% water, this assumption is reasonable. Moreover, the average freezing times of the water and agarose cryogels, obtained experimentally and shown in Figure 9, confirm this assumption. Here, the average freezing time was calculated as the difference between the time when the crystallization began and the time when the sample was completely frozen.

## Figures and Tables

**Table 1:** Notation table.

Variable	Unit of Measurement	Description
ρ	kg/m^3^	Fluid density
$C_{p,ph1}$	J/kg	Specific heat capacity of the solid phase
$C_{p,ph2}$	J/kg	Specific heat capacity of the liquid phase
$C_{p}$	J/kg	Specific heat capacity
$Q_{c}$	J/(m^3^·s)	Power per unit volume due to the latent heat of fusion
$Q_{n}$	J/(m^3^·s)	Power per unit volume due to nucleation
$T^{*}$	K	Temperature of subcooled solution
$T_{f}$	K	Temperature of fluid freezing front
$T_{inter}$	K	Interface temperature between the mold and the agarose solution
$k_{i}$	kg/(m^3^·s)	Nucleation rate per unit volume
$\theta_{ph1}$	-	Volume fraction of the solid phase
$\theta_{ph2}$	-	Volume fraction of the liquid phase
$\kappa_{ph1}$	J/(m·K·s)	Thermal conductivity of the solid phase
$\kappa_{ph2}$	J/(m·K·s)	Thermal conductivity of the liquid phase
$\rho_{ph1}$	kg/m^3^	Density of the solid phase
$\rho_{ph2}$	kg/m^3^	Density of the liquid phase
*T*	K	Temperature
p	Pa	Pressure
t	s	Time
u	m/s	Fluid velocity
I	-	Identity matrix
K	Pa	Stress tensor
g	m^2^/s	Gravitational acceleration
h	m	Height
q	J/(m^2^·s)	Conductive heat flux
κ	J/(m·K·s)	Thermal conductivity
λ	J/kg	Latent heat due to phase change
μ	Pa·s	Fluid dynamic viscosity
$\mu_{T}$	Pa·s	Turbulent fluid dynamic viscosity
k	m^2^/s^2^	Turbulent kinetic energy
ε	m^2^/s^3^	Turbulent kinetic dissipation rate

## Data Availability

The data presented in this study is contained within the article.
